# Economic Status and Decision-Making Behavior: The Cost of Cognitive Control

**DOI:** 10.3390/bs16101877

**Published:** 2026-10-12

**Authors:** Jiachen Lu, Jinting Qiu, Yiwen Li, Yue Cao, Chunyu Li, Yafei Dou

**Affiliations:** 1 School of Education, Guangzhou University, Guangzhou 510006, China; ljcps@gzhu.edu.cn (J.L.);; 2 School of Law, Shenzhen University, Shenzhen 518055, China

**Keywords:** economic disadvantage, cognitive control, decision-making behavior, scarcity theory, contextualism

## Abstract

Economic poverty entails material deprivation and life unpredictability, imposing heavier external constraints on low-income individuals’ goal pursuit than on their higher-income counterparts. We hypothesized that relative economic disadvantage fosters situation-driven decision-making among lower-income groups, which consumes cognitive resources and may impair performance in other cognitive domains. Two experiments were conducted. Experiment 1 (*n* = 41) used a pre/post Stroop task design around a product choice task to examine cognitive control changes; Experiment 2 (N = 903, online) examined decision-making tendencies using a similar product choice task. In Experiment 1, income percentile was marginally and negatively associated with post-task cognitive-control decline (i.e., lower-income participants tended to show greater decline) (*β* = −0.31, *p* = 0.05) and was not significantly correlated with decoy susceptibility (*ρ* = −0.23, *p* = 0.15); the directions of both associations were consistent with our predictions. Because the sample was small and the study was explicitly exploratory, these results should be regarded as preliminary rather than conclusive. Experiment 2 showed that lower-income participants showed significantly higher decoy susceptibility (*β* = −0.22, *p* < 0.001) and significantly slower decisions (*β* = −0.24, *p* < 0.001). Taken together, the well-powered results of Experiment 2, together with the directionally consistent pattern of Experiment 1, indicate that economic disadvantage is associated with greater reliance on external contextual cues and greater cognitive expenditure during decision-making. Because Experiment 2 did not include a direct measure of cognitive control, whether such decision styles in turn impair subsequent cognitive control awaits direct testing.

## 1. Introduction

Economic conditions encompass individual or family resources, including income, assets, and overall wealth, often quantified by metrics such as relative disposable income within specific socioeconomic groups (Jenkins, 2017). As wealth disparities widen globally, these disparities impact decision-making and personality traits (Prinz et al., 2014; Kumar et al., 2023). Lower-income individuals, constrained by limited resources, often prioritize immediate financial concerns, showing strong short-term biases and risk aversion. This affects their consumption, interpersonal relationships, and psychological well-being, particularly in adolescence (Aren & Nayman Hamamci, 2020). Such economic disparities erode psychological resilience, increase stress-induced aggression, and lead to cautious economic behavior among disadvantaged groups. Conversely, higher-income individuals are less risk-averse and more inclined to invest (Bajtelsmit & VanDerhei, 1997), highlighting the profound influence of economic conditions on behavioral patterns and values.

Building on Kahneman’s (1973) “limited cognitive resources theory”, Shah et al. (2012) put forward the “Scarcity Theory” to clarify how financial scarcity—i.e., the lack of monetary resources—exerts an impact on cognition: it forces individuals to concentrate intensely on the management of immediate financial needs (such as covering daily expenses or repaying short-term debts), which in turn depletes their cognitive capabilities. The “Lucky Wheel” experiment demonstrated this, as participants were randomly divided into resource-rich and resource-poor conditions (i.e., given more versus fewer in-game resources), and those with fewer resources subsequently performed worse on cognitive tasks measuring attention and cognitive control, suggesting impaired attention allocation under scarcity. Further evidence from a game-based experiment using “Angry Birds” supported this, where the ‘poor’ group exerted more effort yet performed poorly, especially in tasks involving future planning. Extending this research, Mani et al. (2013) explored economic stress’s impact on cognition in low-income groups. They found that after simulating high-stress economic conditions, cognitive performance deteriorated, a finding corroborated by a field study among sugarcane farmers in India: the same farmers showed lower performance on a cognitive-control (Stroop-type) task and on a fluid-intelligence (Raven’s matrices) measure before harvest, when they were financially strained, than after harvest, when they were relatively better off. These studies collectively indicate that economic scarcity not only diverts necessary cognitive resources but also exacerbates cognitive load, undermining long-term planning and reinforcing a cycle of cognitive limitation in impoverished individuals. Of note, this literature mostly concerns acute, experimentally induced scarcity, whereas chronic economic disadvantage may affect cognition through longer-term changes in habits and decision strategies; we return to this distinction in the General Discussion.

However, in recent years, empirical studies have offered new scrutiny and supplements to the core hypothesis that “scarcity directly impairs cognitive control.” Through empirical audits and replication experiments of 20 scarcity priming studies, O’Donnell et al. (2021) found that only four studies successfully replicated the original effect, and none of them were directly related to cognitive function. In most online experiments, the impact of scarcity manipulations on cognitive control was weak and unstable, suggesting that early laboratory conclusions may have contextual limitations and overestimated effects. Haushofer and Salicath (2023) pointed out that the influence of scarcity on cognitive function needs to distinguish between “anticipated resource fluctuations” and “unanticipated resource shocks”—only unanticipated economic hardships significantly impair cognitive control, while anticipated resource changes have a negligible impact. Through two experiments, Hughes et al. (2024) discovered that chronic financial stress was significantly associated with decreased inhibitory control (measured by the Stop-Signal Task) and visual working memory, but the impact of momentary financial stress was not significant. Additionally, socioeconomic status (SES) exerted a moderating effect: individuals with high SES exhibited a more pronounced decline in cognitive control under momentary financial stress, whereas individuals with low SES showed no such vulnerability, potentially due to long-term adaptation. Collectively, these studies reveal that the impact of economic scarcity on cognitive control is closely related to the “persistence” and “predictability” of scarcity, as well as individuals‘ subjective stress experiences. Therefore, it is necessary to move beyond the simplified framework that manipulating scarcity will inevitably affect cognitive function (Essiz et al., 2023; Manocha et al., 2023; Aren & Nayman Hamamci, 2023).

Poverty, as a persistent and long-term state, extends far beyond the immediate effects of short-term circumstances. Many individuals experience poverty from childhood through adulthood, and sometimes throughout their entire lives. This prolonged hardship significantly shapes individuals’ stable traits and behavioral patterns (Prinz et al., 2014; Hart et al., 2008; Troller-Renfree et al., 2022). Consequently, the influence of economic conditions on individuals is better characterized as a “cultural influence.” People from different economic backgrounds often develop distinct values, habits, and attitudes toward life (Carrera, 2012; De Bruijn & Antonides, 2022).

In research on social class, Kraus et al. (2012) propose that individuals across different social strata gradually develop distinct cognitive tendencies, driven by disparities in material resources and social status. Specifically, those from lower social classes—who frequently face financial scarcity (a core feature of prolonged poverty) and live with persistent uncertainty—tend to rely more on external factors and others’ judgments when making decisions. This reliance fosters a “contextualist” cognitive tendency (Chilman, 1966; Owen et al., 2023; Brady, 2023), which compels them to consider an extensive range of external information during decision-making. While this orientation may seem to promote rational choices on the surface, excessive attention to external cues depletes cognitive resources, ultimately impairing their performance on other cognitive tasks (Kraus et al., 2012). In contrast, individuals from higher social classes—endowed with abundant resources and elevated social standing—prioritize internal factors when pursuing goals, thereby cultivating a “solipsistic” cognitive tendency.

**Figure 1:**
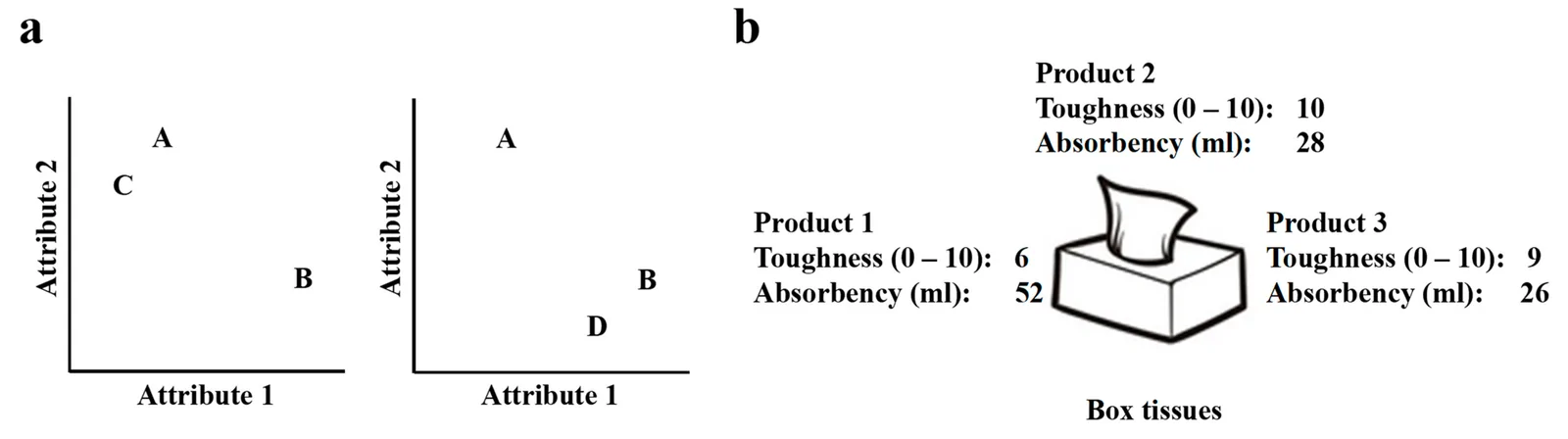
Illustration of choice set configurations inducing the attraction effect (**a**) and example trials from the experiment (**b**). (**a**) The choice sets consist of options A and B, which represent a trade-off between two favorable attributes, and decoy options C and D. When participants are given a choice between A, B, and C, they usually opt for A. However, when offered a choice between A, B, and D, the preference shifts toward B. The structure of this choice set design is adapted from Farmer et al. (2017). (**b**) Examples of typical trials in the product choice task. Participants were required to select one product from three options (taking box tissues as an example here). Among the options, “Product 2” is the target item, “Product 1” is the competitor item, and “Product 3” is the decoy item.

To investigate cognitive resource changes in decision-making contexts, we utilized the “Product Choice Task” from Farmer et al. (2017) alongside the Stroop task (MacLeod, 1991; Chuangchai & Raymaekers, 2023; Garcia-Marques & Fernandes, 2026) to establish situational contexts and assess cognitive shifts. Theoretically, an individual’s preference, such as choosing salmon over steak, should not alter with the addition of a third option like frog legs. However, real-life decision-making reflects greater complexity. Choices are influenced not merely by independent evaluations but significantly by the availability of alternatives (Farmer et al., 2015; Trueblood et al., 2014; Noguchi & Stewart, 2014). As an example of the Product Choice Task adapted from Farmer et al. (2017) (see Figure 1a), consider two target USB drives: one with lower capacity but longer lifespan (Option A) and another with higher capacity but shorter lifespan (Option B). When a decoy option (Option C)—which is dominated by Option A (i.e., slightly worse than A on both attributes) but is better than B on one attribute—is added to the choice set, it is never itself a rational choice; nonetheless, its presence makes A appear more attractive and shifts choices toward A (the attraction effect). Conversely, if an alternative decoy (Option D) is positioned to be slightly inferior to B but superior to A in one aspect, choices shift toward B. This dynamic illustrates how the comparative context of options shapes decisions, highlighting the intricacies of cognitive resource allocation in decision-making scenarios.

We hypothesize that, compared to individuals with higher economic status, those with lower-income status may be more dependent on situational information from their external environment, thus making them more susceptible to the influence of decoy options. This heightened susceptibility leads to greater cognitive resource consumption during decision-making. Specifically, lower-income individuals are likely to exhibit a stronger contextual orientation in their decision-making processes, rendering them more prone to the impact of decoy options. In contrast, higher-income individuals, being less reliant on external situational cues, may consume fewer cognitive resources in such decision-making scenarios. This differential reliance on external cues—with lower-income individuals relying more heavily—depletes their cognitive resources to a greater extent, resulting in a more pronounced decline in performance on subsequent Stroop tasks. These findings would provide evidence of the long-term cognitive depletion effects associated with economic disadvantage.

## 2. Experiment 1

### 2.1. Materials and Methods

#### 2.1.1. Participants

We conducted an a priori power analysis using G*Power 3.1.9.7 (Faul et al., 2009) for a linear regression model with one continuous predictor. Using the large effect size (f^2^ ≈ 0.20; corresponding to Cohen’s d = 0.88–0.94) reported in a comparable study examining income-related cognitive differences (Mani et al., 2013), with α = 0.05 and power = 0.80, the analysis indicated that approximately 39 participants would be sufficient to detect an effect of this magnitude. This served as a planning benchmark rather than a guarantee of adequate power for smaller effects. We recruited 41 healthy undergraduate students (15 males, 26 females; mean age = 20 years, SD = 2.71). Participants’ ages ranged from 18–24 years. Eligibility (including “healthy” status) was verified through a brief self-report screening at recruitment: normal or corrected-to-normal vision, no color blindness or other color-vision impairment, and no history of mental illness. Gender was self-reported and coded as male or female; age was recorded in years. The statistical sensitivity of this sample is characterized by the sensitivity analysis reported in the Results section. Because the large-effect benchmark used above exceeds the effects typically observed in individual-difference research on decision making, it was treated as an upper-bound planning value; given that smaller, more realistic effects could not be reliably detected with this sample size, Experiment 1 is explicitly framed as exploratory. Data were collected through a combination of computer-based tasks and questionnaires administered in a laboratory setting. This research protocol was approved by the Ethics Committee of our University. Prior to participation, all participants provided written informed consent with a clear understanding of the study procedures and their right to withdraw at any time without repercussions—though the specific aims of the study were not disclosed. Participants received a $3 cash reward upon completing the task. All data were anonymized to protect participant privacy.

#### 2.1.2. Experimental Task

**Stroop Task**

**Figure 2:**
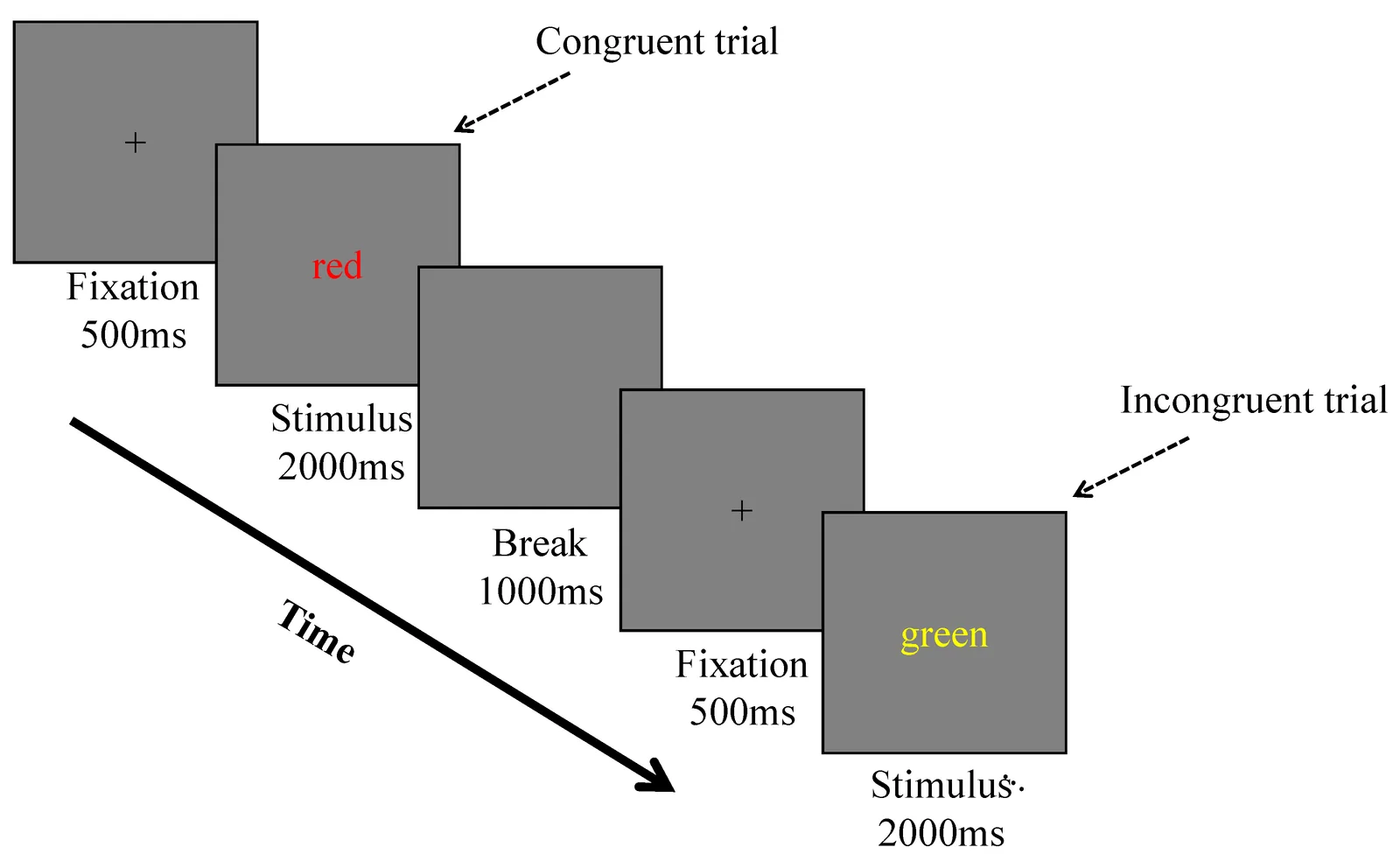
Schematic illustration of the Stroop task procedure. Each trial began with a fixation cross presented for 500 ms, followed by a color word stimulus (e.g., “red” or “green”) displayed for 2000 ms. Participants were required to identify the physical color of the word (red, green, yellow, or blue) while ignoring its semantic meaning. A blank break of 1000 ms preceded the next trial. Congruent trials (word meaning matching its physical color) and incongruent trials (word meaning mismatching its physical color) were randomly intermixed, each accounting for 50% of all trials.

With reference to the study by Job et al. (2013), at the start of the task (as shown in Figure 2), a fixation point was presented on the screen for 500 ms, indicating that subsequent stimuli were about to appear. After the fixation point disappeared, word stimuli were displayed on the screen for 2000 ms. During this period, participants were required to identify the color of the words (red, green, yellow, blue) rather than their semantic meaning. The color words were randomly paired with colors: 50% of the trials were congruent (e.g., the word “red” displayed in red), and the other 50% were incongruent (e.g., the word “red” displayed in blue). Participants were instructed to press specific keys corresponding to the physical color of the words: red for the “D” key, green for the “F” key, yellow for the “J” key, and blue for the “K” key. After participants pressed the corresponding key, they proceeded to the next trial, with an inter-trial interval of 1000 ms.

In incongruent trials, the word’s meaning interfered with the process of naming its physical color, which required cognitive control to inhibit such interference (MacLeod, 1991). Thus, a smaller difference in reaction time between congruent and incongruent trials indicated better cognitive control ability in participants (Job et al., 2013).

Participants first completed 10 practice trials with feedback: after each practice trial, the accuracy of their response was presented on the screen for 1 s, and upon completion of all 10 practice trials, the overall accuracy rate was provided. Only participants who achieved an accuracy rate of 80% or higher in the practice phase were eligible to proceed to the main experimental phase; otherwise, they were required to complete another 10 practice trials.

**Product Choice Task**

The product selection task was adapted from previous studies (Noguchi & Stewart, 2014). Participants were presented with seven pairs of products, and each pair differed across two dimensions (Figure 1a). Each product pair was presented twice: once with a decoy targeting one product, and once with a decoy targeting the other product. Each trial displayed a triad consisting of a target product, a competitor product, and a decoy. The extent to which participants were influenced by the environment was measured by observing the frequency of their selections of the target product, while the cognitive effort they invested was examined through the average response time of their choices.

Each trial was presented independently in the center of the computer screen (as shown in Figure 1b). Participants were instructed to select a product by pressing specific keys: the “N” key for the left product, the “J” key for the top product, and the “M” key for the right product. There was no time limit for each trial, and the next trial began immediately after a selection was made. The positions of the three products were randomly assigned, and the trial order was also randomized. However, product categories were not repeated until all seven product pairs had been presented.

Notably, to simulate a more realistic scenario, the experimenter verbally informed participants that this task involved “new products to be launched by Yuguo Supermarket” (a fictional name). They were told the supermarket was the sponsor of the experiment and requested participants to help test product preferences while completing the experiment. Participants were instructed to make selections from three product options based on both personal preferences and economic status.

**Experimental Procedure**

Participants sat in front of a computer and completed the following four task segments in sequence:

(1) Stroop Task (Pre-test): Participants first completed a Stroop task comprising 72 test trials preceded by at least 10 practice trials; each trial presented a color word for up to 2000 ms, followed by a 1000-ms inter-trial interval. Including instructions, practice with accuracy feedback, and self-paced responding, the pre-test block took approximately 20 min in total. This task was designed to assess their cognitive control abilities prior to the product choice task. Upon completion of the Stroop task, participants took a 1-min break.
(2) Product Choice Task: After the break, participants completed a 10-min product choice task. Following a familiarization session to ensure proficiency, they proceeded to the official task, which consisted of two blocks. Each block contained 14 trials involving seven product sets. The products in the second block did not overlap with those in the first block, and the difference between the decoy products and the target products was smaller (see Supplementary Materials for specific product types and details of the differences). A 1-min break was provided between the two blocks.
(3) Stroop Task (Post-test): After completing the product choice task, participants rested for a 2-min interval before completing a second Stroop task. This task consisted of 72 official trials, with the aim of assessing participants’ cognitive control abilities following the product choice task. Including instructions and practice, this post-test block took approximately 18 min in total.
(4) Economic Status Questionnaire: As the final part of the experiment, participants completed a questionnaire regarding their family and personal economic status. The questionnaire included items about the household per capita monthly income in the past year, with participants required to input the corresponding amounts independently.

#### 2.1.3. Data Analysis Strategy for Experiment 1

To preserve statistical power and avoid the loss of information associated with extreme-group dichotomization, we treated household per capita monthly income as a continuous predictor in all primary analyses. Because the income distribution was positively skewed, we converted raw income values into percentile ranks (based on the full sample, *n* = 41) to reduce the influence of outliers and facilitate interpretability of regression coefficients.

For the Stroop task, we used hierarchical multiple regression to examine whether income predicted post-task cognitive control performance after accounting for baseline (pre-task) performance. In Step 1, we entered pre-task ∆RT (the reaction-time difference between incongruent and congruent trials) as a control variable. In Step 2, we entered income percentile as the predictor of interest. The dependent variable was post-task ∆RT. This approach directly tests whether income adds incremental predictive value beyond individuals’ initial cognitive control ability. A parallel regression was conducted for accuracy data (∆ACC) to rule out speed–accuracy trade-off interpretations. Additionally, to verify that baseline cognitive control did not differ by income level, we conducted a simple regression predicting pre-task ∆RT from income percentile.

For the product choice task, given the small sample size and the exploratory nature of the decision-making indices, we computed Spearman rank-order correlations (due to non-normal distributions) between income percentile and two key indices: the decoy-induced index (frequency of choosing the target product when a decoy was present) and mean decision response time.

All statistical analyses were performed using SPSS 22 (IBM, Armonk, NY, USA), with *α* = 0.05. Due to the limited sample size (*n* = 41), we interpret results with appropriate caution, emphasizing effect magnitudes rather than relying solely on null-hypothesis significance testing (cf. Cumming, 2014). Effect sizes are reported as standardized regression coefficients (*β*) and Spearman’s *ρ*.

### 2.2. Results

#### 2.2.1. Stroop Task Performance: Hierarchical Regression Analysis

Baseline comparison. To confirm that income levels did not differ in initial cognitive control, we first regressed pre-task ∆RT on income percentile. Results showed that income percentile did not significantly predict pre-task ∆RT, *β* = 0.15, *p* = 0.35, *R*^2^ = 0.02, indicating that baseline cognitive control was comparable across income levels. This finding justifies the subsequent hierarchical regression examining post-task performance while controlling for baseline differences.

**Figure 3:**
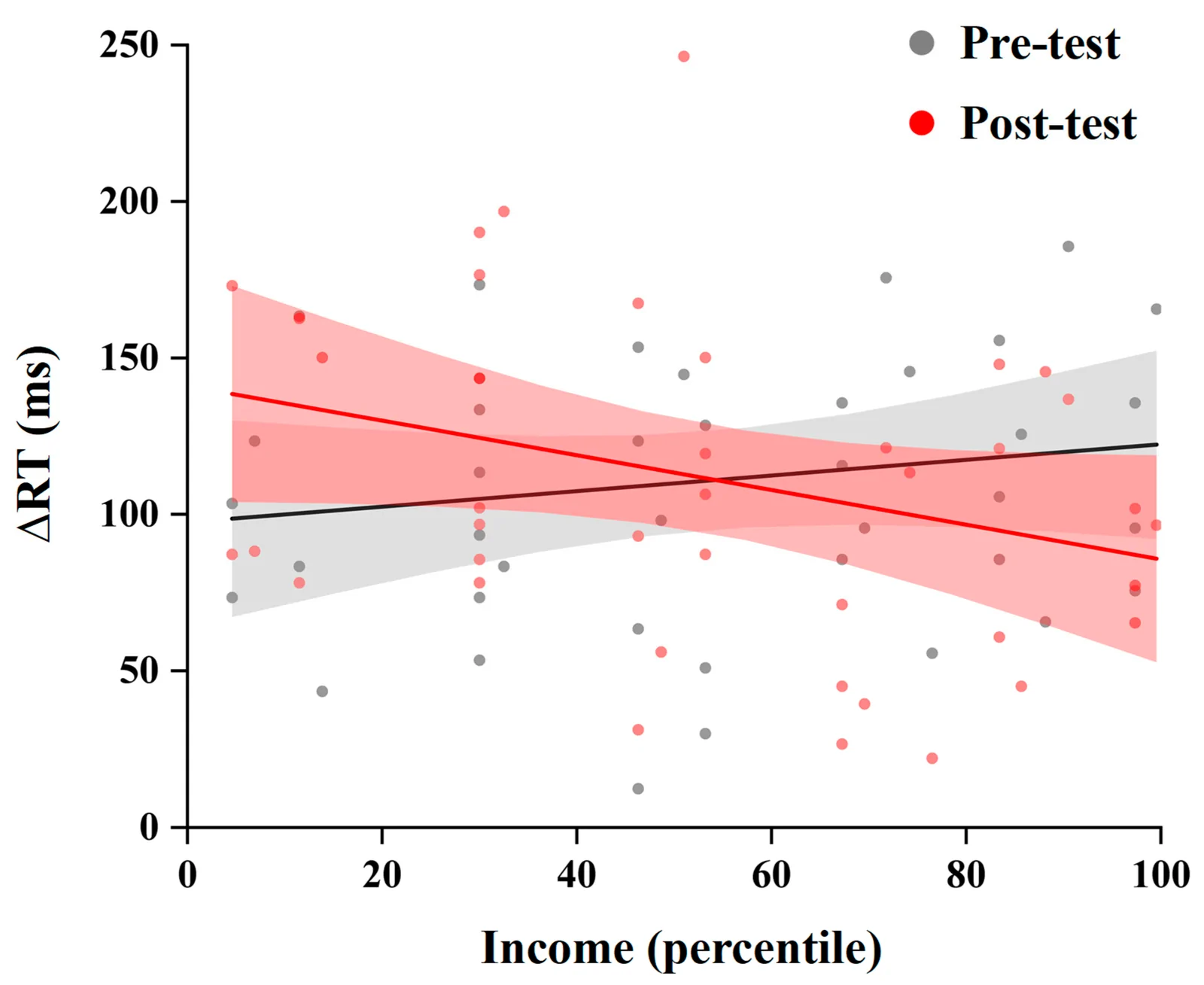
Scatter plot showing the relationship between household income percentile and Stroop interference (∆RT) in Experiment 1 (*n* = 41). Gray circles represent pre-task Stroop performance; red circles represent post-task Stroop performance. Solid lines represent linear fits with 95% confidence intervals (shaded areas).

We then examined whether income percentile predicted post-task cognitive control (∆RT) after controlling for baseline performance. In Step 1, pre-task ∆RT did not significantly predict post-task ∆RT, *F*(1, 39) = 0.71, *p* = 0.40, *R*^2^ = 0.02, indicating that individual differences in baseline cognitive control did not simply carry over to the post-task measurement. In Step 2, adding income percentile increased the explained variance, Δ*R*^2^ = 0.09, Δ*F*(1, 38) = 3.98, *p* = 0.05 (two-tailed), indicating a marginally significant incremental effect at the conventional 0.05 level. In the final model, income percentile negatively predicted post-task ∆RT, suggesting that participants with higher economic status tended to show better cognitive control (i.e., smaller Stroop interference) after the product choice task, even after adjusting for their pre-task performance (see Figure 3). Given the small sample and the exploratory nature of this study, we interpret this marginally significant, directionally consistent pattern cautiously as preliminary and in need of replication in a larger, better-powered sample.

Supplementary power analysis. To contextualize the statistical power of Experiment 1, we conducted a post-hoc sensitivity analysis using G*Power (Faul et al., 2009) for a linear regression model with one predictor (*n* = 41, *α* = 0.05, power = 0.80). The analysis indicated that the sample was adequately powered to detect only large effects (*f*^2^ ≈ 0.20), but not small-to-medium effects. The observed effect size for income percentile predicting post-task ∆RT (*β* = −0.31, corresponding to *f*^2^ ≈ 0.11) fell below this threshold.

To confirm that baseline accuracy did not differ by income level, we first regressed pre-task ∆ACC on income percentile. Results showed that income percentile did not significantly predict pre-task ∆ACC, *β* = −0.01, *p* = 0.89, *R*^2^ < 0.001, indicating that baseline response accuracy was comparable across income levels. To examine whether the observed ∆RT effects might reflect a speed–accuracy trade-off, we then conducted a parallel hierarchical regression analysis predicting post-task accuracy (∆ACC) from pre-task ∆ACC (Step 1) and income percentile (Step 2). Pre-task ∆ACC did not significantly predict post-task ∆ACC in Step 1, *F*(1, 39) = 0.01, *p* = 0.93, *R*^2^ < 0.001. Adding income percentile in Step 2 did not improve the model, Δ*R*^2^ < 0.001, Δ*F*(1, 38) = 0.04, *p* = 0.85. In the final model, neither pre-task ∆ACC nor income percentile was a significant predictor. These results indicate that income-related differences were specific to interference-based reaction-time measures (∆RT) rather than overall response accuracy, thus ruling out a simple speed–accuracy trade-off interpretation of the primary findings.

#### 2.2.2. Product Choice Task Performance: Exploratory Correlational Analyses

To explore the relationship between economic status and decision-making behavior in the laboratory setting, we computed Spearman rank-order correlations between income percentile and two key indices from the product choice task: the decoy-induced index (frequency of choosing the target product when a decoy was present) and mean decision response time. We used Spearman’s ρ given the small sample size and the non-normal distribution of the decision indices (Shapiro–Wilk tests, *p*s < 0.05).

**Figure 4:**
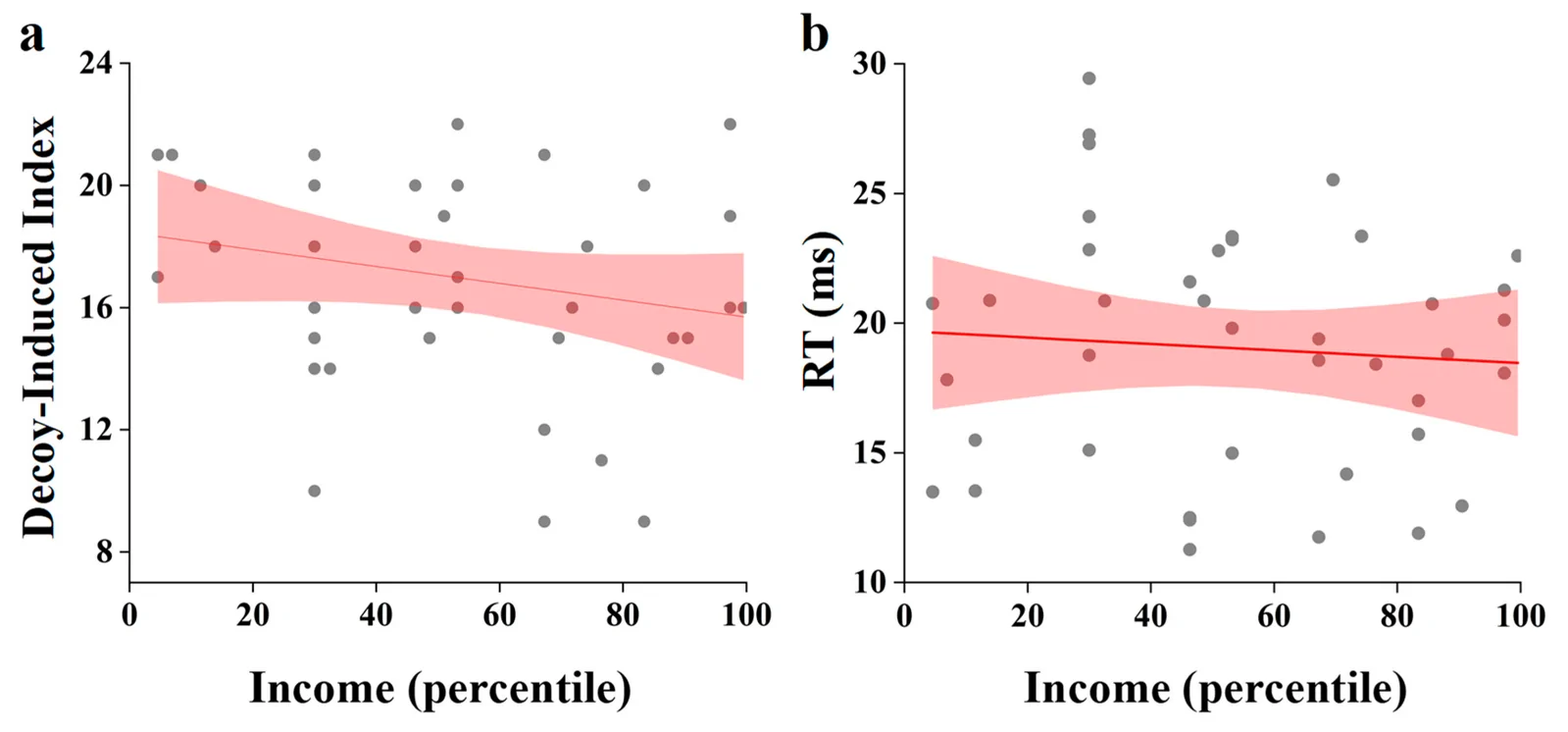
Scatter plots of household income percentile against (**a**) decoy-induced index and (**b**) mean response time in the product choice task of Experiment 1 (*n* = 41). Solid lines show linear fits with 95% confidence intervals (shaded areas).

As shown in Figure 4, income percentile was not significantly correlated with the decoy-induced index, *ρ* = −0.23, *p* = 0.15, nor with response time, *ρ* = −0.07, *p* = 0.67. The correlation between the decoy-induced index and response time was also not significant, *ρ* = −0.09, *p* = 0.58, suggesting that these two decision indicators tapped relatively independent aspects of the choice process. Taken together, none of the correlations involving income reached statistical significance in Experiment 1; the income–decoy correlation was directionally consistent with our prediction, but the sample was too small to detect such effects reliably (see the sensitivity analysis above).

Although the correlation between income and the decoy-induced index did not reach conventional significance, its direction was consistent with our hypothesis (higher income less decoy susceptibility), and the effect size (*ρ* = −0.23) falls within the small-to-medium range by conventional benchmarks (Cohen, 1992). Given the limited statistical power associated with the sample size of Experiment 1 (*n* = 41)—which was sufficient to detect only large effects (*ρ* ≈ 0.43) at 80% power—this statistically non-significant correlation does not provide evidence against the hypothesized relationship, but rather underscores the need for a larger, better-powered investigation. This motivated the design of Experiment 2, which used a substantially larger online sample to re-examine the income–decision-style link with adequate statistical sensitivity.

### 2.3. Discussion

The results of Experiment 1 revealed no significant association between income percentile and baseline cognitive control, as indicated by both reaction time and accuracy measures, suggesting that baseline cognitive performance was comparable across levels of economic status. After completing the product choice task, income percentile showed a marginally significant negative association with post-task cognitive control in the predicted direction (i.e., lower economic status tended to be associated with greater cognitive-control decline). This marginally significant, directionally consistent pattern is consistent with our hypothesis that economic status plays a role in cognitive depletion during decision-making, although, given the small sample, it should be interpreted cautiously.

With regard to the product choice task itself, income percentile was not significantly correlated with either the decoy-induced index or response times. The absence of significant associations in decision-making indicators may be attributable to several factors. First, the limited sample size in Experiment 1 constrained statistical power to detect small-to-medium effects, which are typical in decision-making research (Aber & Fonseca, 2014). Second, the laboratory setting might have influenced participants’ decision-making strategies, potentially leading them to overemphasize product differences or adopt more rational approaches than they would in real-life situations (McCambridge et al., 2012). Third, the repetition of product choices across trials may have encouraged participants to rely on memory-based habitual selections (Gasbarri et al., 2014) rather than engaging in deliberate evaluation of each option, thereby reducing the task’s sensitivity to capture nuanced income-related decision patterns.

Although the decoy-induced index did not show a statistically significant correlation, it is worth noting that the direction of the effect was consistent with our hypothesis. This directional consistency, together with the directionally consistent post-task cognitive-control pattern, is consistent with—but does not establish—the theoretical account that lower-income individuals may consume more cognitive resources during context-dependent decision-making. However, given the exploratory nature of these findings and the methodological constraints discussed above, the conclusions drawn from Experiment 1 should be interpreted with appropriate caution.

The modest sample size of Experiment 1, as reflected in the sensitivity analysis reported above, limits the generalizability of these findings. This power limitation, together with the potential artificiality of the laboratory environment, motivated the design of Experiment 2. By employing a substantially larger online sample, Experiment 2 aimed to re-examine the relationship between economic status and decision-making tendencies with improved statistical sensitivity and greater ecological validity, thereby providing a more robust test of our core hypotheses (Bruch & Feinberg, 2017).

## 3. Experiment 2

### 3.1. Materials and Methods

#### 3.1.1. Participants

We used GPower software (Faul et al., 2009) to estimate the required sample size for a linear regression model with one continuous predictor. Based on a comparable decision-making study (Farmer et al., 2017) that reported small-to-medium effects in similar product choice tasks, we assumed a conservative effect size of *f*^2^ = 0.02 (corresponding to *R*^2^ ≈ 0.02; Cohen, 1988). With α = 0.05 and power = 0.80, the analysis indicated that 395 participants would be required. Note that this benchmark was adopted from published decision-making research rather than from Experiment 1, because Experiment 1’s small sample (*n* = 41) was adequately powered only to detect large effects and its observed effects were not statistically reliable; using Experiment 1 as the sole basis for the power calculation would therefore have been inappropriate. A total of 949 responses were collected via the SurveyPlus online platform from participants across mainland China. The sample was not limited to university students but included individuals from a wide range of occupational backgrounds, such as corporate employees, self-employed professionals, and other working adults. After excluding 46 responses due to incomplete data or extreme response times (mean RT < 2 s or >32 s), 903 valid datasets were retained. Among these participants, there were 414 males and 489 females, with a mean age of 21.70 years (SD = 3.33), ranging from 17 to 51 years. The survey did not include separate attention-check items, and quality control consisted solely of the exclusions described above. Gender was self-reported and coded as male or female. As in Experiment 1, age and gender were not included as covariates in the primary analyses because of the study’s focus on income and the sample’s relatively narrow age range; we return to their potential role in the General Discussion.

The research protocol was approved by the Ethics Committee of our University. Prior to completing the questionnaire, all participants were fully informed of the study procedures, as well as their right to withdraw from the study at any time without incurring adverse consequences. To avoid potential response biases, the specific objective of the study was not disclosed to participants. Furthermore, all responses and data were processed anonymously to safeguard the privacy and confidentiality of participants’ information. Participants were provided with a $0.5 reward upon the completion of the questionnaire.

#### 3.1.2. Online Product Choice Task

The online version of the Product Choice Task, adapted from Farmer et al. (2017), was used in Experiment 2. Experiment 2 utilized 18 distinct product categories. In each category, participants were presented with three options: Option A, Option B, and either Option C or Option D. For 9 categories, the decoy (C) was designed to have two inferior attributes compared to Product A. For the remaining 9 categories, the decoy (D) was designed to have two inferior attributes compared to Product B. This design aimed to assess how the presence of different types of decoys influenced participants’ decision-making behavior and cognitive resource consumption.

#### 3.1.3. Experimental Procedure

The experiment was administered via the SurveyPlus online platform and consisted of 19 pages in total. The first page included two parts: collection of participants’ personal information (e.g., age, gender, and household per capita monthly income over the past year), and presentation of the instructions and informed consent form. The form stated: “Welcome to Yutean Supermarket’s product survey. You are asked to select products based on your own preferences and needs. We will analyze your selections to guide our purchasing decisions. This survey will take approximately 10 min, during which you will need to make choices across 18 product categories. Participation is voluntary; all information will be kept anonymous, and you will receive compensation after completing the survey”. Pages 2 to 18 were dedicated to the product selection task. On each of these pages, participants were presented with one product category and asked to select the product they preferred from three options. After making their choice, participants automatically proceeded to the next page. The platform recorded the time spent on each page and the final product selection, providing the data for subsequent analysis.

#### 3.1.4. Data Analysis for Experiment 2

Consistent with Experiment 1, we treated income as a continuous predictor using percentile ranks (computed from the full sample) in all main analyses. We conducted linear regression analyses to examine whether income percentile predicted (a) the decoy-induced index (frequency of choosing the target product in the presence of a decoy) and (b) decision response time. Because response time was positively skewed, we log-transformed it (Log_RT = LN(Response_Time)) prior to analysis. This approach uses the full range of income data and avoids the loss of information associated with extreme-group comparisons. All analyses were performed using SPSS 22, with *α* = 0.05, and effect sizes are reported as standardized *β* coefficients. In addition to the primary models, we also conducted supplementary regression analyses with age and gender entered as covariates to examine the robustness of the observed income effects.

### 3.2. Results

**Figure 5:**
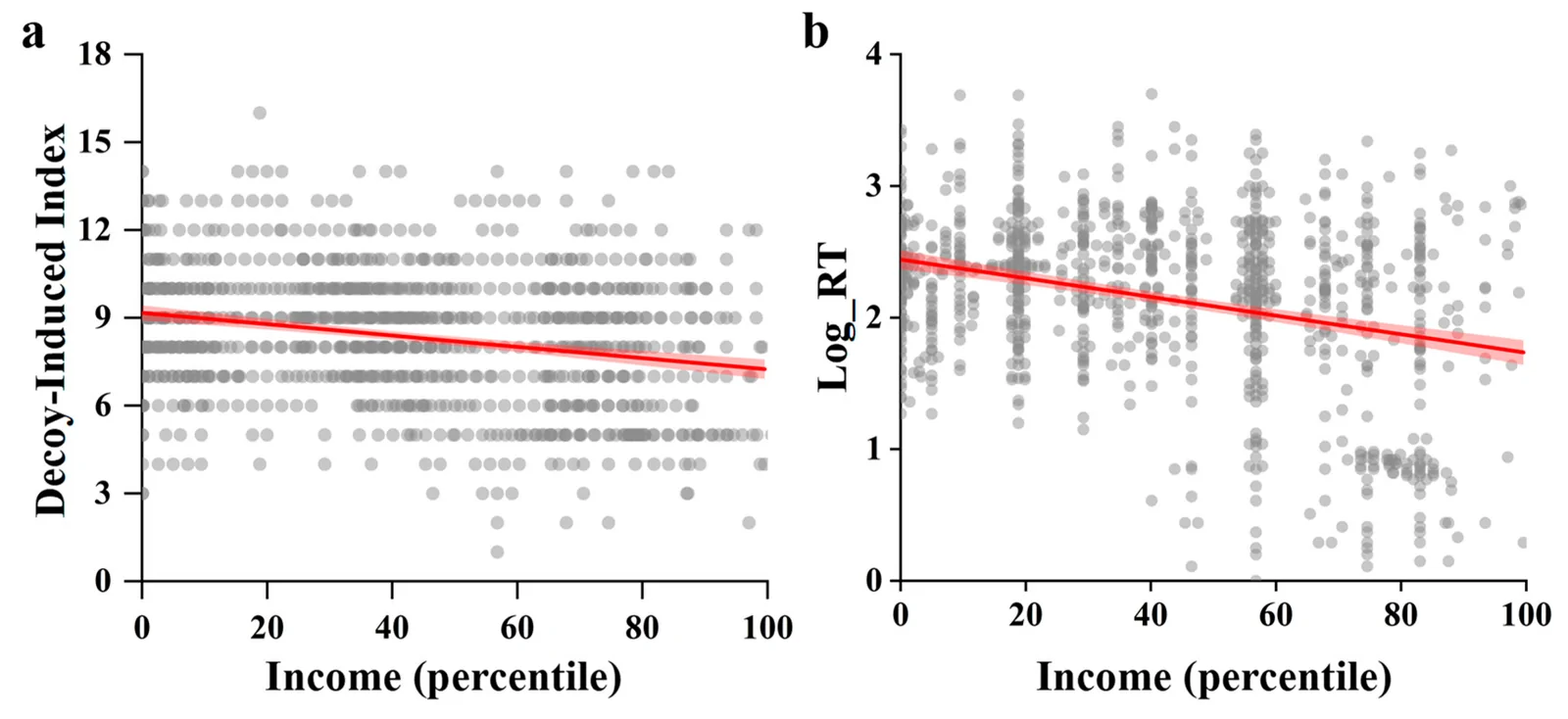
Scatter plots of household income percentile against (**a**) decoy-induced index and (**b**) log-transformed response time (Log_RT) in Experiment 2 (*n* = 903). Solid lines show linear fits with 95% confidence intervals (shaded areas).

To examine whether the relationships between income and decision-making indicators remained robust after controlling for age and gender, we conducted multiple linear regression analyses with age and gender entered as covariates. In the first model, age did not significantly predict the decoy-induced index (*β* = −0.04, *p* = 0.29), whereas gender did (*β* = 0.09, *p* = 0.01). Critically, income percentile significantly negatively predicted the decoy-induced index even after controlling for these demographic variables (*β* = −0.22, *p* < 0.001), indicating that lower-income participants remained more susceptible to the influence of decoy options during product choice (see Figure 5a). In the second model, neither age (*β* = 0.03, *p* = 0.32) nor gender (*β* = 0.02, *p* = 0.69) significantly predicted log-transformed decision response time, whereas income percentile remained a significant negative predictor (*β* = −0.24, *p* < 0.001), suggesting that lower-income individuals took longer to make decisions, consistent with higher cognitive effort during the choice process, independent of age and gender (see Figure 5b). Overall, the overall models were significant for both outcomes: *F*(3, 899) = 17.39, *p* < 0.001, *R*^2^ = 0.06 for the decoy-induced index, and *F*(3, 899) = 18.71, *p* < 0.001, *R*^2^ = 0.06 for log-transformed response time. These results confirm that the observed income effects on decision-making were not attributable to age or gender differences.

These results, obtained with a large and more ecologically valid online sample, are consistent with the exploratory pattern observed in Experiment 1 and provide a well-powered test of the income–decision-style relationship. They indicate that economic disadvantage is associated with greater reliance on external contextual cues (i.e., decoy susceptibility) and increased cognitive expenditure during decision-making.

### 3.3. Discussion

Experiment 2, conducted via an online survey with a large valid sample, provided critical empirical support for the study’s core hypothesis while addressing key limitations of the laboratory-based Experiment 1. First, income percentile negatively predicted the decoy-induced index, providing well-powered evidence that lower economic status is associated with a stronger contextual orientation in decision-making. This aligns with Kraus et al.’s (2012) theoretical proposition that economic scarcity leads lower social classes to rely more heavily on external situational cues—here, lower-income participants were more susceptible to the influence of decoy options when evaluating products, whereas higher-income participants showed greater resistance to such biases, likely relying on internal preferences or stable evaluation criteria instead. Second, income percentile negatively predicted decision response time, indicating that lower-income individuals took longer to make decisions—a pattern consistent with greater cognitive effort during the choice process. However, because Experiment 2 did not include a direct measure of cognitive control (e.g., a Stroop task), it provides only indirect evidence about cognitive expenditure; claims about downstream effects on cognitive control therefore rest primarily on the exploratory results of Experiment 1 and require direct testing. Together, these results suggest that economic disadvantage is associated with greater reliance on external contextual cues and increased cognitive expenditure during decision-making, and they set the stage for—but do not by themselves demonstrate—the post-decision cognitive-control pattern observed in Experiment 1.

Experiment 2’s design also enhances the study‘s validity and generalizability. Unlike Experiment 1’s laboratory setting—where demand characteristics (e.g., participants adjusting strategies to align with perceived experimenter expectations) might have distorted decision processes—the online format more closely mirrors real-life contexts (e.g., online shopping with multi-option product displays), reducing artificial task effects. The large sample size overcomes the power limitations of Experiment 1 and provides more robust estimates of the income–decision relationship. Additionally, these results contribute to ongoing debates about Scarcity Theory (O’Donnell et al., 2021; Haushofer & Salicath, 2023): by focusing on relative economic status (rather than temporary scarcity priming) and domain-specific situational decision tasks (rather than abstract cognitive tests), they provide evidence that economic disadvantage is associated with decision biases and greater cognitive resource consumption. Overall, Experiment 2 provides well-powered evidence consistent with the pattern suggested (but not established) in Experiment 1, supporting the view that economic status is associated with decision-making patterns characterized by differential reliance on external situational cues and corresponding cognitive resource expenditure.

With respect to the distribution of decision times in Figure 5b, the observations near the upper portion of the income distribution (approximately the 80th percentile) that show comparatively low decision times reflect a subset of participants who completed the product choices especially quickly. These fast deciders tended to report relatively higher household incomes, and their short decision latencies are consistent with the overall negative income–decision-time association reported above; part of this association therefore reflects a subgroup of higher-income participants who made their selections quickly rather than deliberating at length. In addition, because self-reported household incomes clustered at round values (e.g., multiples of 1000 RMB), many participants share the same income-percentile rank, which produces dense vertical bands in the scatter plot; within each such band, decision times vary around the band’s average, which may contribute to the appearance of symmetry around particular percentile values. These features of the data distribution reflect coarse, round-number reporting of household income and genuine variability in decision speed rather than an artifact of the analyses.

## 4. General Discussion

This study systematically explored the effects of economic status on cognitive control and decision-making through two experiments. Experiment 1 showed a marginally significant negative association between income percentile and post-decision cognitive-control decline and a non-significant correlation with decoy susceptibility; both effects were in the predicted direction, but the small sample warrants a cautious interpretation. Experiment 2, using a large online sample, provided well-powered evidence for the hypothesized decision-style differences: lower-income participants showed significantly higher decoy susceptibility and significantly slower decisions. Taken together, these two studies provide convergent—though not direct mediating—evidence that economic disadvantage shapes decision-making by increasing reliance on external contextual cues and consuming cognitive resources, and they suggest—but do not directly demonstrate—that this may in turn impair subsequent cognitive control.

### 4.1. Analyzing from the Perspective of Scarcity Theory: The Chain Effects of the Low-Income Group on Cognition and Decision-Making

The Scarcity Theory posits that deficits in economic resources force individuals to prioritize immediate survival needs, leading to significant depletion of cognitive resources (Mani et al., 2013; De Bruijn & Antonides, 2022; Ridley et al., 2020; Attanasio et al., 2022). This depletion impairs complex decision-making and long-term planning, increases cognitive load, and is particularly pronounced in multitasking scenarios (Dean et al., 2017). Economic stress not only presents external material challenges but also affects psychological states and decision-making styles, driving individuals to focus on short-term needs at the cost of long-term goals (De Bruijn & Antonides, 2022; Sheehy-Skeffington, 2020).

Consistent with these findings, our research indicates that while lower-income individuals initially exhibit cognitive control comparable to their higher-income counterparts, their cognitive control deteriorates significantly after engaging in resource-intensive tasks such as product selection. This post-task cognitive fatigue and resource depletion hinder subsequent task performance, reinforcing a vicious cycle between economic disadvantage and inefficient decision-making. Such conditions exacerbate the adverse consequences of economic hardship, confirming the profound psychological and cognitive impacts of financial scarcity (Ong et al., 2019; Pepper & Nettle, 2017).

Notably, participants in the current study were recruited from university settings in major first-tier cities in China, and their reported household incomes, while spanning a wide range, were generally above the country’s official poverty line ($105). As a result, our sample did not include individuals experiencing extreme material deprivation. Nevertheless, we still observed a meaningful association between income percentile and post-task cognitive performance, suggesting that the cognitive consequences of economic disadvantage are not confined to absolute poverty but extend along the income continuum. This finding aligns with prior research indicating that poverty and economic hardship are not merely absolute conditions but also multidimensional phenomena closely tied to social context and relative socioeconomic standing (Duclos & Gregoire, 2002; Ravallion & Chen, 2019). Thus, even moderate differences in relative economic status within a non-poor sample can exert detectable effects on cognitive functioning in resource-demanding contexts.

### 4.2. Potential Mechanisms Underlying Post-Decision Cognitive Control Decline in Low-Income Groups

Based on the combined results of the two experiments, the potential mechanisms underlying the post-decision cognitive control decline in lower-income groups can be explored from multiple perspectives. First, it may be related to the consumption of cognitive resources during decision-making. As proposed in relevant theoretical frameworks (Baumeister et al., 2007), tasks that require continuous evaluation and comparison of product attributes and the influence of decoys (such as the product choice task) can occupy limited cognitive resources, leaving fewer resources available for subsequent cognitive control tasks like the Stroop task. For lower-income individuals, who tend to rely more on external situational cues when making decisions (Kraus et al., 2012), this resource consumption may be more pronounced, ultimately leading to poorer post-task performance.

However, alternative explanations centered on motivational and attentional processes also merit consideration (Inzlicht et al., 2014; Lurquin & Miyake, 2017). The observed differences in cognitive control may not stem solely from resource depletion but rather from shifts in task priorities after decision-making. After expending effort on the product choice task, individuals may be more inclined to prioritize “want-to” goals (e.g., ruminating on financial concerns or desired products) over “have-to” goals (e.g., focusing on the requirements of the Stroop task). For lower-income participants, such preoccupations related to economic circumstances or product preferences may be more salient, reducing the working memory capacity allocated to the subsequent cognitive control task and thus impairing performance. This aligns with recent research emphasizing that motivational and attentional trade-offs, rather than just resource limits, shape post-task cognitive performance (Inzlicht et al., 2014; Lurquin & Miyake, 2017).

It should be noted that the aforementioned mechanisms are not mutually exclusive. The impact of economic status on post-decision cognitive control may involve both the consumption of cognitive resources during decision-making and motivational shifts in task priorities. For instance, the greater reliance on external cues among lower-income individuals may simultaneously increase cognitive resource use and strengthen the tendency to prioritize “want-to” goals after the task, jointly contributing to the observed decline in cognitive control. Future research could further adopt methods such as concurrent physiological measurements (e.g., glucose monitoring) or attentional tracking (e.g., eye-tracking) to disentangle the relative contributions of these mechanisms.

### 4.3. Economic Disadvantage’s “Cognitive Tax”: Evidence from Non-Economic Pressure Scenarios and Its Impact on Decision-Making

This study, without direct economic pressure as all products were identically priced, revealed that lower-income individuals were more susceptible to the influence of a decoy option, suggesting cognitive depletion even in non-pressured scenarios (Ridley et al., 2020; Mesler et al., 2022; Destin & Svoboda, 2018). Participants from lower-income backgrounds demonstrated slower, less efficient decision-making processes, underscoring that economic disadvantage affects not just material conditions but also cognitive function and decision-making abilities. Exposure to economic scarcity leads these individuals to prioritize immediate concerns over long-term benefits, impairing their decision-making efficiency and ability to address complex socio-economic challenges effectively (De Bruijn & Antonides, 2022; Herd & Walsemann, 2024). The findings highlight a “cognitive tax” imposed by economic disadvantage, subtly undermining decision-making capabilities and exacerbating social inequality. This study confirms the significant cognitive burden of economic disadvantage, emphasizing its deep impact on individual decision-making beyond immediate survival, contributing to a cycle of entrenched disadvantage.

It is important to emphasize that more thorough, context-sensitive deliberation is not inherently detrimental. Comparing options carefully and attending to the immediate choice environment can improve decision quality and may reflect an adaptive, maximization-oriented strategy (Schwartz et al., 2002). The present findings should therefore not be read as claiming that lower-income individuals decide “worse” because they deliberate more. Rather, our argument concerns the cognitive cost of such deliberation: when a decision maker must make many such choices or faces competing cognitive demands, exhaustive comparison of external cues consumes limited attentional resources and may leave fewer resources available for subsequent tasks (Schwartz et al., 2002). Whether effortful deliberation helps or harms therefore depends on the context, the stakes, and the number of decisions—a nuance that future research should examine directly.

### 4.4. Policy Implications: Addressing Poverty’s Cognitive Burden in Alleviation Strategies

This study underscores the necessity for policymakers to account for the cognitive control impact of economic disadvantage when designing alleviation strategies, highlighting that economic disadvantage not only reduces economic stability but also depletes cognitive resources, affecting decision-making (Lavner et al., 2015; Phan & Collins, 2019). Traditional poverty reduction measures primarily focus on economic support like increasing income or subsidies, often neglecting the cognitive burdens faced by lower-income individuals. Findings indicate that these individuals are more susceptible to external influences, with slower and less efficient decision-making processes, primarily due to cognitive resource depletion. This cognitive tax—an invisible psychological burden—can hinder their ability to benefit from policies and services effectively. To counter this, policies should simplify administrative procedures, reduce application complexities, and provide cognitive support through decision aids and guidance, thus alleviating the cognitive load and enabling better decision-making and resource utilization among disadvantaged groups.

In terms of personal decision-making, and drawing on the study‘s conclusions, lower-income individuals may reduce cognitive load through structured approaches: setting clear decision goals in advance to minimize distractions and impulsive choices, breaking complex tasks into smaller steps to ease stage-specific cognitive pressure, and seeking advice from family or professionals to alleviate burdens—particularly for important decisions, as social support lowers cognitive demands in resource-scarce contexts (Zhong et al., 2022; Betkó et al., 2022). It should be noted that this study has not directly verified the effectiveness of these strategies for lower-income groups, and their validity requires further testing in specific scenarios. However, based on the research consensus that “resource scarcity affects decision-making by occupying cognitive resources and altering task priorities” (De Bruijn & Antonides, 2022), structured strategies may offer insights for coping with decision dilemmas by reducing resource waste and optimizing attention allocation. Meanwhile, behavioral economics research emphasizes that interventions targeting resource-scarce groups must align with their cognitive load characteristics to avoid overburden from overly complex strategies (DellaValle, 2019; Aren & Nayman Hamamci, 2023)—suggesting that future research should explore the adaptability of these strategies in specific contexts.

### 4.5. Limitations and Future Directions

Several caveats should be noted. First, the sample in Experiment 1 was modest (N = 41). As reported in the results section, a post-hoc sensitivity analysis indicated that this sample was adequately powered to detect only large effects, which are considerably larger than the effects typically observed in individual-difference studies of decision-making. The critical regression coefficient was only marginally significant and was smaller than the benchmark used for the power analysis. Therefore, this finding should be treated as exploratory and requires replication in larger samples. We have reported confidence intervals and emphasized the preliminary nature of this result throughout the manuscript. Second, although Experiment 2 provided a well-powered test of decision-making differences, it did not include a cognitive control post-test, so we cannot directly test the full mechanistic chain within a single study. Third, our participants were university students and community respondents in mainland China; their household incomes, while ranging widely, do not represent extreme poverty. Thus, our conclusions are best generalized to relative economic disadvantage rather than absolute poverty. Fourth, the online format of Experiment 2, while ecologically valid, limited our control over participants‘ attention and environmental distractions—a common limitation of web-based research (e.g., Oppenheimer et al., 2009). Nonetheless, the fact that we obtained significant effects under these conditions underscores the robustness of the observed income–decision-style relationship. Fifth, the age range of our samples was limited (Experiment 1: university students, mean age ≈ 20 years; Experiment 2: mean age = 21.70 years, range 17–51 years). Choice consistency and susceptibility to context effects such as the attraction (decoy) effect can vary across the adult lifespan, with older adults sometimes showing reduced susceptibility to such context effects relative to younger adults (Kim & Hasher, 2005; Faust et al., 2006), and with decision quality developing and changing with age more generally (Brocas et al., 2019). Older decision makers would also have experienced economic (dis)advantage for far longer than the young adults in Experiment 1, so the present data cannot speak to the cumulative effects of lifelong disadvantage. Relatedly, because age and gender were not included as covariates in the primary models, future work with more age-diverse samples should examine their potential confounding role. Sixth, our income measure captured household per-capita monthly income over the past year, so the design cannot distinguish chronic from recent or recurrent economic disadvantage; chronic and acute scarcity may have different cognitive consequences (O’Donnell et al., 2021; Haushofer & Salicath, 2023), and future research should assess the duration and trajectory of financial hardship. Seventh, the cognitive assessment in these studies was limited: only Experiment 1 included a direct measure of cognitive control (the Stroop task), and Experiment 2 measured decision time as an indirect proxy of cognitive effort. Future research should include well-validated assessments of specific cognitive-control components (e.g., Stroop and flanker interference tasks, stop-signal tasks, and working-memory tasks such as N-back) to determine which abilities are affected by economic disadvantage and to test the full decision-to-cognition chain within a single study. Eighth, in Experiment 1, Stroop interference was operationalized as a difference score between incongruent and congruent trials. This composite index does not by itself indicate whether any effect originates in incongruent (interference) or congruent (facilitation) trials; future work should report condition-level reaction times and accuracies so that the locus of any income-related difference can be established.

Future work should employ longitudinal designs or experimental manipulations of scarcity to establish causality, incorporate physiological measures (e.g., pupillometry, EEG) to directly index cognitive effort during decision-making, and recruit samples from more economically diverse populations to test the generalizability of these findings. In addition, longitudinal designs that track individuals across changing economic circumstances, together with samples spanning late adulthood, would help disentangle the effects of chronic versus acute disadvantage and of age-related differences in decision style (Brocas et al., 2019; Kim & Hasher, 2005).

## 5. Summary

To address how economic status influences decision-making and cognitive control—specifically whether relative economic disadvantage is associated with situation-driven decision tendencies and cognitive depletion—this study conducted two complementary experiments. The laboratory experiment tracked pre- and post-decision cognitive control changes, while the larger-sample online survey analyzed decision-making indicators to verify situational tendencies and cognitive consumption. Together, they overcame single-method limits (laboratory precision, online generalizability), and the well-powered findings of Experiment 2, together with the directionally consistent results of the exploratory Experiment 1, indicate that economic status is associated with context-dependent decision behavior and cognitive expenditure; whether this impairs subsequent cognitive control requires direct testing. Taken together, the results are broadly consistent with the initial research question—whether relative economic disadvantage shapes decisions by increasing reliance on external cues (e.g., susceptibility to decoys) and consuming cognitive resources—and they reinforce the theoretical link between economic disadvantage and decision-related cognitive costs.

## Data Availability

The data and analysis scripts for all studies have been made publicly available via OSF and can be accessed at https://osf.io/9yp4r (accessed on 6 October 2026). All studies, measures, manipulations, participant inclusion/exclusion criteria, and data exclusions employed in this research are fully reported in the main text of the manuscript. Relevant supporting data (e.g., specific product types, detailed differences between stimuli, and supplementary statistical analyses) have been deposited in the Supplementary Material to ensure methodological transparency and reproducibility. No unreported studies, measures, or data processing procedures were involved in the development of this manuscript.

## Supplementary Materials

The following supporting information can be downloaded at: https://www.mdpi.com/article/10.3390/bs16101877/s1, Table S1: The products used in the product choice task within the first block of Experiment 1; Table S2: The products used in the product choice task within the second block of Experiment 1; Table S3: Products Used in the Product Choice Task of Experiment 2; Figure S1: Regarding the example of the “sports watch”.
