# Supplementary material for: Economic Status and Decision-Making Behavior: The Cost of Cognitive Control
Source: Behav Sci (Basel). 2026 Oct 12;16(10):1877. doi: 10.3390/bs16101877 (PMC13701578; doi:10.3390/bs16101877)
Supplement: Supplementary file 1 [file behavsci-16-01877-s001.zip › Product Choice Task in Experiment 1.pdf]

Table S1. In Experiment 1, the products used in the product choice task within the first block. the products used in the product choice task within the first blockDecoy option C is designed to induce preference for Option A; Decoy option D is designed to induce preference for Option B.

Item	Attribute	A	B	C	D
USB Drive	Capacity (GB)	16	32	12	28
	Lifespan (Months)	36	20	32	16
Tissue Paper	Toughness (0-10)	4	8	3	7
	Water Absorption Capacity (ml)	52	28	46	22
Orange Juice	Vitamin C (mg)	34	82	22	70
	Calories (Kcal)	33	69	42	78
Printer	Cost (RMB per Page)	0.2	0.1	0.3	0.15
	Speed (Pages per Second)	17	6	13	2
Headphones	Sound Quality (0-100)	68	92	62	86
	Lifespan (Months)	24	12	20	10
Highlighter	Brightness (0-1)	0.4	0.8	0.3	0.7
	Capacity (ml)	180	100	160	80
Sports Shoes	Durability Lifespan (Months)	8	32	2	26
	Comfort Level (0-100)	88	64	82	58

Table S2. In Experiment 1, the products used in the product choice task within the second block. the products used in the product choice task within the first blockDecoy option C is designed to induce preference for Option A; Decoy option D is designed to induce preference for Option B.

Item	Attribute	A	B	C	D
Car	Safety Rating (0-5)	4	5	3.8	4.8
	Fuel Efficiency (Kilometers per Liter)	50	31	44	25
Internet Service Provider	Average Speed (kb/s)	386	646	290	550
	Download Limit (GB)	292	150	270	128
Six-Pack Beer	Price (RMB)	7	3	7.5	3.5
	Taste (0-5)	4	2.5	3.8	2.2
Mobile Phone Battery	Price (RMB)	175	120	190	135
	Talk Time (Hours)	14	11	13	10
Light Bulb	Price (RMB)	1.2	2.4	1.5	2.7
	Lifespan (Hours)	1000	1800	980	1980
Mouthwash	Freshness Duration (Hours)	6	12	5	11
	Capacity (ml)	25	16	23	14
TV	Screen Size (Inches)	40	65	38	63
	Clarity (0-10)	6.23	4.63	5.5	3.9
