# Supplementary material for: Economic Status and Decision-Making Behavior: The Cost of Cognitive Control
Source: Behav Sci (Basel). 2026 Oct 12;16(10):1877. doi: 10.3390/bs16101877 (PMC13701578; doi:10.3390/bs16101877)
Supplement: Supplementary file 1 [file behavsci-16-01877-s001.zip › Product Choice Task of Experiment 2.pdf]

Table S3. Products Used in the Product Choice Task of Experiment 2. Decoy option C is designed to induce a preference for target Option A, while decoy option D is designed to induce a preference for target Option B.

| Product Category | Attribute | A | B | C | D |
|---|---|---|---|---|---|
| Mobile Phone (Apps) | Number of Installable Apps | 20 | 30 | | 28 |
| | Number of Simultaneously Running Apps | 6 | 4 | | 3 |
| External Hard Drive | Capacity (TB) | 4 | 6 | 3.5 | |
| | Transfer Speed (mb/s) | 1000 | 600 | 900 | |
| Tissue Paper (Boxed) | Toughness (0-10) | 8 | 12 | | 11 |
| | Water Absorption Capacity (ml) | 52 | 28 | | 26 |
| Orange Juice | Vitamin C (mg) | 34 | 82 | | 80 |
| | Taste (0-10) | 8.5 | 5.3 | | 5 |
| Speaker | Power (w) | 35 | 52 | | 50 |
| | Signal-to-Noise Ratio (db) | 86 | 95 | | 90 |
| Highlighter | Brightness (0-10) | 0.4 | 0.8 | 0.3 | |
| | Capacity (ml) | 180 | 100 | 160 | |
| Sports Shoes | Durability Lifespan (Months) | 5 | 24 | 8 | |
| | Comfort Level (0-100) | 82 | 64 | 88 | |
| TV | Resolution (px) | 4k | 8k | 3k | |
| | Color Gamut (NTSC) | 1 | 0.85 | 0.95 | |
| Desktop Computer | CPU Performance (0-100) | 75 | 100 | | 93 |
| | GPU Rendering Performance (0-100) | 95 | 60 | | 70 |
| Sports Watch | Function Index (0-5) | 3.2 | 5 | 3 | |
| | Standby Time (h) | 168 | 96 | 150 | |
| USB Flash Drive | Capacity (GB) | 32 | 64 | | 60 |
| | Transfer Speed (mb/s) | 500 | 300 | | 280 |
| Tissue Paper (Rolled) | Toughness (0-10) | 4 | 8 | 3 | |
| | Water Absorption Capacity (ml) | 52 | 28 | 50 | |
| Wine | Smoothness (0-10) | 3.4 | 8.2 | 3.2 | |
| | Taste (0-10) | 8.5 | 5.3 | 7.9 | |
| Bluetooth Headphones | Sound Quality (0-10) | 7.1 | 9.1 | 6.8 | |
| | Standby Time (h) | 12 | 3.2 | 11 | |
| Neutral Pen | Smoothness (0-10) | 6.1 | 8.2 | | 7.9 |
| | Capacity (ml) | 180 | 100 | | 110 |
| Slippers | Durability Lifespan (Months) | 8 | 16 | | 15 |
| | Comfort Level (0-100) | 67 | 44 | | 41 |
| Projector | Brightness (ANSI) | 600 | 1000 | | 900 |
| | Color Gamut (NTSC) | 1 | 0.85 | | 0.82 |
| Laptop | CPU Performance (0-100) | 75 | 95 | 72 | |
| | GPU Rendering Performance (0-100) | 9 | 6 | 8 | |

Product 3：
Functionality index (0–5) 3.2
Standby time (h) 168

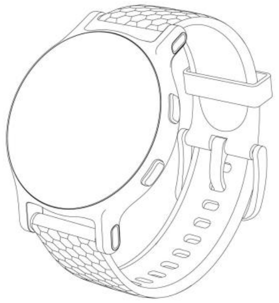

Product 1：
Functionality index (0–5) 5
Standby time (h) 96

Product 3：
Functionality index (0–5) 3
Standby time (h) 150

Sports Watch

Figure S1: Regarding the example of the "sports watch". Participants were required to select one option from Product 1, Product 2, and Product 3. Options A, B, C (D) were randomly assigned to Products 1, 2, and 3; in other words, the positions of Options A, B, C (D) were randomized.
